# Assessment of Periodontitis Risk Factors in Endodontically Treated Teeth: A Cross-Sectional Study

**DOI:** 10.3390/diagnostics14171972

**Published:** 2024-09-06

**Authors:** Mihaela Sălceanu, Cristina Dascălu, Anca Melian, Cristian Giuroiu, Cristina Antohi, Corina Concita, Tudor Hamburda, Claudiu Topoliceanu, Maria-Alexandra Mârţu

**Affiliations:** 1 Department of Odontology-Periodontology and Fixed Restorations, Faculty of Dental Medicine, University of Medicine and Pharmacy “Grigore T. Popa”, 700115 Iasi, Romania; mihaela.salceanu@umfiasi.ro (M.S.); giuroiu.cristian@umfiasi.ro (C.G.); cristina.antohi@umfiasi.ro (C.A.); corina-alexandra.concita@umfiasi.ro (C.C.); tudor.hamburda@umfiasi.ro (T.H.); claudiutopoliceanu@yahoo.com (C.T.); maria-alexandra.martu@umfiasi.ro (M.-A.M.); 2 Discipline of Medical Informatics and Biostatistics, Faculty of Dental Medicine, University of Medicine and Pharmacy “Grigore T. Popa”, 700115 Iasi, Romania

**Keywords:** endodontically treated teeth, endo-periodontal lesions, periodontitis, prevalence, risk factors, radiographic diagnosis

## Abstract

The aim of the study was to collect data about the prevalence and risk factors of apical periodontitis in a population of endodontically treated patients. The study group included 151 patients (52 males, 99 females; mean age 48.36 ± 15.708 yrs.) with 391 endodontically treated teeth (mean follow-up of 5.25 ± 1.759 yrs.). According to the initial tooth diagnosis, root-filled teeth were divided into Group A, root-filled teeth treated for pulpitis or for the purpose of prosthetic pulpectomies (vital pulp group), and Group B, root-filled teeth with non-vital pulp (necrotic pulp). Clinical and radiographic evaluation of the root and its periapical area were performed to establish the success/failure of endodontic therapy, the quality of the root canal fillings (length, density, taper), and coronal restoration. The presence of recurrent caries, periodontal pathology, or endo-periodontal lesions were also recorded. Univariate and multivariate analyses were used to determine the risk factors for apical periodontitis and calculate their odds ratios (ORs). For the root-filled vital pulp tooth group, the highest risks for apical periodontitis are associated with inadequate homogeneity (OR 30.938), periodontitis (OR 9.226), and over-filling (OR 8.800). For the root-filled non-vital pulp tooth group, the highest risks are associated with periodontitis (OR 4.235) and age over 60 yrs. (OR 4.875). For the necrotic pulp tooth group, multivariate analysis identified an age > 60 yrs., filled molars, intracanal posts, poor coronal restoration quality, under-filling, and periodontitis as significant combined risk factors. Inadequate root canal filling and periodontitis in both groups were risk factors associated with most cases of apical periodontitis. Other risk factors include age > 60 yrs., poor coronal restoration quality, and the presence of intracanal posts in root-filled teeth with necrotic pulp.

## 1. Introduction

The uncontrolled spread of pulp inflammation can progress to more advanced pulpitis, and potentially involving the apical tissues and requiring extirpation to alleviate pain, followed by nonsurgical root canal treatment to preserve the health of the apical tissues [1]. While pulp necrosis without microbial contamination often goes unnoticed, eventually pulpal necrosis and infection lead to apical inflammation, necessitating nonsurgical root canal treatment to manage the infection in the endodontic space. Early and accurate diagnostic may be challenging in this stage. Also, root canal treatment may be required for treatment of vital teeth as part of a prosthodontic plan or in cases of noncontaminated pulp necrosis resulting from traumatic dental injuries [2].

Apical periodontitis is the result of the complex processes generated by microbial factors penetration into apical areas and host defense reactions as a result of this invasion of the peri-radicular tissues [3]. Apical periodontitis can result from teeth with necrotic pulp or endodontically treated teeth and is an important cause of reoccurrence of endodontic infection and subsequent tooth extraction. Teeth with failed endodontic treatment present bacterial biofilm next to or at distance from the apical foramen. While intra-radicular biofilm can be effectively cleaned by using combination of ultrasounds and NaOCl 2% or lasers [4], extra-radicular biofilm, resistant to antibacterial agents [5] is an important factor of failed endodontic treatment this biofilm being detected in vitro on the apical external surface in teeth with necrotic pulp and radiographically visible periapical lesions [6].

Various non-surgical techniques [7,8,9,10,11] as well as surgical approach can be used to eliminate the bacterial infection of the endodontic space [12,13]. Asymptomatic periapical lesions in teeth with poor quality endodontic treatments usually harbor anaerobic microorganisms, in similar compositions with that of the infected and untreated teeth [14].

The signs of healing processes following treatment of apical periodontitis are as follows: repair process in apical area, absence of the increase in radiolucency in the peri-radicular areas, biological sealing, or even the presence of a fibrous capsule [15]. Also, a successful endodontic therapy is characterized by the absence of pain, proper endodontic sealing, adequate coronal sealing, as well as the presence of healing periapical processes [16]. Successful outcome of teeth with apical periodontitis ranges from 86% to 98% [17].

Despite the improvement of awareness, knowledge, techniques and materials in endodontic therapy, a 2% rate of annual loss of root-filled teeth [18] proves the relevance of various risk factors contributing to failure (newly developed or persistent periapical radiolucency around a root-filled tooth apex) [19].

Some of the factors influencing the outcome of endodontic treatment include the use of dam, intraoperative procedures (i.e., irrigation method), the extent of apical preparation, the use of antibacterial medications within the canal, as well as the number of sessions required to finish the treatment. However, the results can be impacted by the pre-operative symptoms, severity of bone loss in the apical areas, the anatomical complexity of the canal’s apical area, as well as the virulence and persistence of the bacterial infection [19].

The dynamic of host/infection interaction is influenced both by intra-operative therapeutic factors to sustain a microbial ecological shift and the role of the tooth and its marginal sealing to resist infection recurrence [14]. Regardless of factors implied, apical periodontitis is due bacteria and their byproducts present within the root canal system leading to host’ immune response [20]. The prevalence of failures in the endodontic therapy ranges from 32.8% [21] to 39% [22], 39.5% [23], 41% [24], and 72.1% [25].

The non-surgical treatment is recommended both for medium and severe apical periodontitis as the bone extension of the apical periodontitis is not correlated with the post-treatment evolution of the healing processes [23]. The decisions of monitorization and delay of endodontic treatment, retreatment, or extraction are influenced by the experience and competencies of the practitioner, tooth prosthetic value, aesthetic demands, cost/benefits analysis, patient’ systemic status, as well as the patient preferences [24,25]. The diagnostic of apical periodontitis in root-filled tooth may require retreatment which can decrease the success rate of the endodontic therapy [25], due to presence of resistant biofilm on external radicular surface [25,26].

Risk factors are those variables or conditions that increase the likelihood of a person developing a disease. The onset of diseases is influenced by a multitude of risk factors, which often intersect or overlap with each other [17,27,28,29,30]. These independent variables must be considered in the decision of non-surgical treatment in apical periodontitis. When tooth restorability is feasible, in routine dental practice most dentists and patients agree with the maintenance of a root-filled tooth with apical periodontitis to avoid further dental tissues degradation [17].

The aim of study was to assess and analyze the prevalence and risk factors of apical periodontitis in a population of endodontically treated patients to evaluate and better manage this category of patients in the context of a complex oral rehabilitation.

## 2. Materials and Methods

The study group included 151 patients (52 males, 99 females; mean age 48.36 ± 15.708 yrs.), with 391 endodontically treated teeth (mean follow-up of 5.25 ± 1.759 yrs.). The patients attended a private dental clinic for the first time between the years 2014 and 2022. This study adhered to the principles outlined in the Declaration of Helsinki and was approved by the Ethics Department of the University of Medicine and Pharmacy “Grigore T. Popa” (Nr. 334/16.07.2023) in Iasi. All patients participating in the study provided informed consent by signing a consent form approved by the ethics committee of the University of Medicine and Pharmacy “Grigore T. Popa” in Iasi. The inclusion criteria were as follows: root-filled teeth with at least 24 months post-treatment; root canal fillings following pulpectomy (pulpitis, prosthetic pulpectomies) or endodontic treatment for necrotic pulp; all root-filled teeth had healthy periapical status (PAI 1, according to the Orstavik periapical index) [31] at the moment of the initiation of endodontic therapy; teeth with a composite resin filling or full coverage crown. The exclusion criteria were as follows: a systemic pathology that can negatively influence periapical healing (uncontrolled diabetes as well as its complications, osteoporosis, rheumatoid arthritis, head and cervical chemotherapy or radiotherapy, autoimmune diseases); medication administration (immunosuppressive medication, corticosteroids, bisphosphonates); subjects with teeth that have undergone apical resection.

According to the initial endodontic diagnosis, root-filled teeth were divided into two groups: Group A, vital pulp teeth (teeth treated for pulpitis, prosthetic pulpectomies) (*n* = 127), and Group B, necrotic pulp teeth (*n* = 391).

Table 1 exposes the social and demographic parameters of the subjects, while the study group variables are described in Table 2.

All patients were evaluated by one calibrated experienced clinician (S.M.). The independent variables assessed as risk factors for endodontic therapy failure (presence of apical periodontitis) were as follows: dental groups, location (maxillary/mandibular), coronal restoration type (amalgam/composite fillings, full coverage crown), presence of intracanal posts, the quality of the root-canal fillings, the quality of the coronal restoration marginal sealing, recurrent caries, periodontal status, endo-periodontal lesions, the presence of an opposing tooth.

The Orstavik periapical index (PAI) [31] was used to assess periapical statuses in the study groups. In multi-rooted teeth, the worst PAI score was considered.

Endodontic therapy success was defined by the absence of clinical symptoms, radiographic images with rarefaction of the periapical area, and a periodontal ligament with normal width or that was slightly widened [32].

Failure of the endodontic therapy was defined by the clinical signs and symptoms of apical inflammation, widening of the periodontal ligament, or the presence of any periapical radiolucency with PAI > 2 [32].

The statuses of the root-canal fillings and coronal restoration marginal sealing were assessed as ‘adequate’ or ‘poor’ using the radiographic criteria shown in Table 3 [25,31].

The root-canal filling length was measured on periapical radiographs to the nearest 0.1 mm. The assessed parameters of the endodontic treatment were as follows: length (adequate; poor); density (adequate; poor); conicity (adequate; poor).

The criteria for the assessment of the quality of the root canal fillings (length, density, taper) as well as the criteria for coronal restoration quality are shown in Table 3. Additionally, any tooth with fractured or absent coronal restoration was classified in the category of poor coronal restoration.

The presence of recurrent caries was diagnosed both in clinical examination and in radiographic images.

Periodontitis of an endodontically treated tooth was clinically diagnosed according to the 2018 AAP/EFP classification of periodontal and peri-implant diseases.

Statistical analysis. The statistical tests were performed in SPSS 29.0 software. Frequency distribution was used to characterize the qualitative variables. Descriptive statistics (mean values, standard deviations) were used to characterize the quantitative variables. The Chi-squared test was used to analyze the relationships between the socio-demographic variables, clinical and radiographic features, and the periapical status; the identified risk factors were characterized by calculating the associated ORs (odds ratios) using a model of univariate binary logistic regression. Their combined action over the periapical status was evaluated further using a multivariate binary logistic regression. The logistic regression models were generated using the Enter method, and their adequacy was evaluated using the Hosmer–Lemeshow goodness-of-fit test. *p* < 0.05 was the threshold for significance; *p* < 0.01 was evaluated as statistically highly significant.

## 3. Results

**Figure 1:**
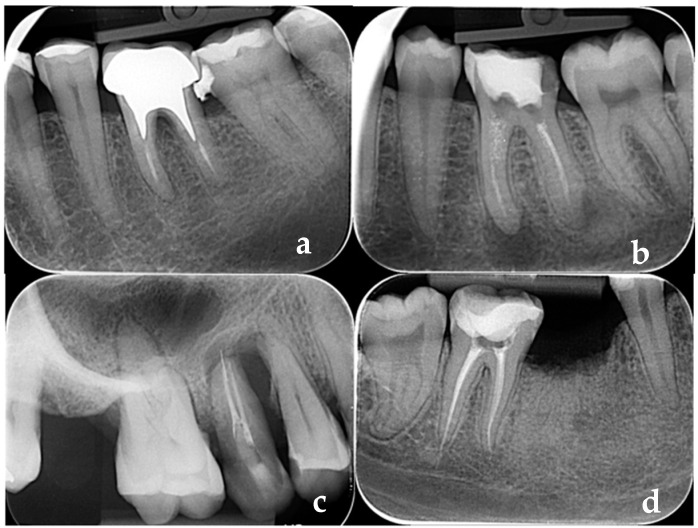
(**a**–**d**) Apical periodontitis in teeth with poor quality root-canal fillings: (**a**,**b**) short fillings; (**c**,**d**) over-fillings.

Apical periodontitis was diagnosed in 35 root-filled teeth in the vital pulp group (27.7% AP prevalence) and 146 teeth in the necrotic pulp group (55.3% AP prevalence). Figure 1a–d. exposes teeth with poor root-canal fillings diagnosed with apical periodontitis. Table 4 exposes the success/failure rates of the endodontic treatment, related to the assessed independent variables, for Group A (vital pulp) and Group B (necrotic pulp). Table 5 shows the univariate and multivariate analysis of the independent variables assessed for risk of apical periodontitis in Group A (vital pulp) and Group B (necrotic pulp).

Figure 1a–d illustrates teeth with poor root-canal fillings diagnosed with apical periodontitis.

The prevalence of apical periodontitis in both study groups is exposed in Table 4. Regarding the effectiveness of the endodontic treatment in relation to gender, AP prevalence was higher in the root-filled teeth with necrotic pulp (52.8%-males, 56.3%-females) when compared with the vital pulp group (20.9%-males, 31%-females). In the necrotic pulp group, root-filled teeth in patients > 60 yrs. had significant higher AP prevalence (81.8%) when compared with other age groups (*p* = 0.024*); significant differences were not found between age groups in the vital pulp tooth group. Regarding the root-filled tooth location, molars (63.4%) had significantly higher AP prevalence than premolars (44.3%) in the necrotic pulp group, while the maxillary/mandibular location was not associated with any significant difference in both groups (*p* > 0.05). The follow-up duration (2–4 yrs. vs. 5–9 yrs.) was not significantly associated with AP prevalence (*p* > 0.05). In the vital pulp tooth group, root-filled teeth reconstructed with composite resin restorations had a higher AP prevalence rate (50%) than those with full coverage crowns (23.4%), with significant statistical difference (*p* = 0.014 *); this difference was not found in the necrotic pulp group (*p* > 0.05). Within necrotic pulp group teeth, reconstruction with intra-canal posts was significantly associated with AP (66.3%) when compared with teeth without intra-canal posts (33.3%) (*p* = 0.007 **). Significant statistical difference (*p* = 0.007 **) in AP prevalence was found in the vital pulp tooth group between root-filled teeth with good-quality coronal restoration (15.8%) and poor-quality coronal marginal sealing (37.1%). In the vital pulp tooth group, 42.1% of root-filled teeth with low-quality root canal fillings were diagnosed with AP, while all teeth with adequate endodontic treatments were diagnosed with healthy periapical status. In the necrotic pulp group, 60.4% of root-filled teeth with inadequate root canal fillings were diagnosed with AP, while only 28.6% of teeth with adequate root canal fillings had apical pathology (*p* ≤ 0.001 **). Related to the categories of technical errors, all types of inadequate root canal fillings (over-fillings, under-fillings, inadequate homogeneity, inadequate taper) lead to significant statistical differences in AP prevalence (in both groups) when compared with adequate root canal fillings (*p* < 0.001 **). Root-filled teeth with caries adjacent to coronal restorations in the necrotic pulp group had significantly higher AP prevalence (60.7%) when compared with those without caries adjacent to coronal restorations (45.8%) (*p* = 0.019 *). Also, teeth with caries adjacent to coronal restorations in the vital pulp group had significantly higher AP prevalence (42.4%) when compared with those without caries adjacent to coronal restorations (11.5%) (*p* < 0.001 **). Periodontal pathology of the root-filled teeth in the necrotic pulp group was associated with significantly higher AP prevalence (74.8%) when compared to periodontally healthy root-filled teeth (41.2%) (*p* < 0.001 **). Also, periodontal pathology of the teeth in the vital pulp group was associated with significant higher AP prevalence (42.5%) when compared to periodontally healthy teeth (7.4%) (*p* < 0.001 **). Root-filled teeth with endo-periodontal lesions had 100% prevalence of apical periodontitis in both groups. The presence of an antagonist tooth in occlusal contact with a root-filled tooth was not associated with apical periodontitis (*p* > 0.05).

Univariate regression analysis showed that presence of composite resin filling, poor coronal restoration quality, inadequate root canal filling quality (under-filling, over-filling, inadequate homogeneity), the presence of an opposing tooth, the presence of caries adjacent to coronal restorations, and presence of periodontitis, for the vital pulp tooth group, were identified as risk factors associated with a higher rate of apical periodontitis (Table 5). The highest risks are associated with inadequate homogeneity (OR 30.938), periodontitis (OR 9.226), and over-filling (OR 8.800). For the vital pulp tooth group, multivariate analysis did not prove other associations. In vital pulp teeth, a combination of the identified risk factors will not significantly influence the presence of apical periodontitis. The presence of these combined risk factors results in a greater chance of apical periodontitis when compared to teeth with adequate coronal restoration, adequate root canal filling quality, and healthy gingival status. Univariate regression analysis in the necrotic pulp group showed that an age over 60 yrs., filled molars, the presence of intracanal posts, poor coronal restoration quality, inadequate root canal filling quality (under-filling, over-filling, inadequate homogeneity), the presence of caries adjacent to coronal restorations or periodontitis, and endo-periodontal lesions were identified as risk factors associated with a higher rate of apical periodontitis. The highest risks are associated with periodontitis (OR 4.235) and an age over 60 yrs. (OR 4.875). For the necrotic pulp tooth group, multivariate analysis identified as significant combined risk factors an age over 60 yrs., filled molars, intracanal posts, poor coronal restoration quality, under-filling, and periodontitis. The presence of these combined risk factors results in a greater chance of apical periodontitis than root-filled teeth treated for necrotic pulp in patients with an age under 60 yrs., filled premolars, adequate coronal restoration, an absence of caries adjacent to coronal restorations, adequate root canal filling quality, and healthy gingival status (Table 5).

## 4. Discussion

Our study analyzed the influence of various potential risk factors on the prevalence of apical periodontitis related to the treatment of teeth with vital pulp (pulpitis, prosthetic pulpectomies) or necrotic pulp. Various systemic conditions (chronic debilitation, uncontrolled diabetes, metabolic disorders, osteoporosis) can decrease the ability of the immune system to heal the periapical tissues and thus increase the failure rate even after proper endodontic treatment [33]. In our study, we aimed to focus on some risk factors that can be controlled by dentists; thus, the presence of systemic pathologies that can be related to disrupted healing processes was an exclusion criterion.

According to logistic regression analysis, socio-demographic variables of the patients with root-filled teeth were not found to be associated with apical periodontitis in root-filled teeth with vital pulp before endodontic treatment, while the age group over 60 yrs. is significantly associated with apical periodontitis in teeth treated for necrotic pulp. Data regarding the relation between the failure of endodontic treatment and sociodemographic parameters are controversial. Frisk et al. (2015) reported that age was found to be a predictor, while gender was not associated with apical periodontitis [34]. Hussain et al. (2024) found gender (females) to be a significant predictor, while age was not associated with the presence of apical periodontitis [35].

Molars had significantly higher AP prevalence than premolars in root-filled teeth treated for pulp necrosis. Their complex anatomy, root canal curvatures, and ramifications complicate both the biochemical cleaning stage and the root canal shaping and filling [36]. Microcracks and even root fractures can occur in teeth where root canals are prepared using multi-rotary devices. However, some multi-file rotary systems have less risk of microcracks than others. Proper selection of a multi-rotary system and endodontist experience can contribute to a significant decrease in tooth fracture risk [37]. Regarding the use of prosthetic crowns versus composite resin fillings, a research group found low quality evidence for the effects of crowns when compared to conventional fillings for the coronal restoration of root-filled teeth [38].

In our study, direct composite restoration of root-filled teeth treated for pulpitis or prosthetic pulpectomies significantly increased the rate of apical periodontitis. Proper marginal sealing is crucial for preventing microleakage and ensuring the longevity of the restoration and endodontic treatment, as it minimizes the risk of caries adjacent to the restorations, especially in Class II composite resin restorations [39,40]. Root-filled teeth restored with indirect restorations have a better survival rate when compared to teeth restored with direct restorations, but the difference was not significant, and the type of coronal restoration was not considered a risk factor for apical periodontitis [17]. However, Dawson et al. (2016) did not find statistical differences between the rates of apical periodontitis in root-filled teeth restored with resin composite, amalgam, or crowns [41].

However, intracanal posts placed to increase retention of composite fillings or complete coverage crowns were found to be risk factors for apical periodontitis both in univariate (OR 2.114) and multivariate analyses (OR 2.177) of the root-filled teeth treated for pulp necrosis. The relation between the presence of an intracanal post and apical periodontitis is controversial. A research group also reported a 16% increase in apical periodontitis rates in root-filled teeth restored with composite resin restorations or complete coverage crowns and intracanal posts [42].

Adequate root canal filling can provide complete sealing in order to hinder the communication between the endodontic space and the periapical tissue [9]. In our study, the quality of root canal fillings (length, homogeneity, taper) was found to be a risk factor of apical periodontitis in univariate and multivariate analyses both for root-filled teeth treated for pulpitis or prosthetic pulpectomies and teeth treated for pulp necrosis. Hussain et al. (2024) also reported that poor root canal fillings had a 7.92 times higher risk of apical periodontitis when compared to root-filled teeth with proper-quality endodontic treatment [35]. We found that root-filled teeth with canal overfilling had an 11.214 times higher risk of the presence of apical periodontitis; teeth with poor density of the root-canal filling had a 4.824 times higher risk of endodontic treatment failure; and short fillings had a 1.733 times higher risk of apical periodontitis when compared to root-filled teeth with adequate root canal fillings. A research group found short fillings to have a higher risk of apical periodontitis (OR 2.77), while canal overfilling had a significantly lower risk of apical periodontitis (OR 1.08) when compared to the result reported by our study [43]. Root canal filling quality was also found to be a predictor of apical periodontitis by Frisk et al. (2015) [34]. Root canal fillings at 1–2 mm distance to apex had significantly higher success rates when compared with root-filled teeth with short fillings and over-fillings [44,45]. Poor homogeneity of the root canal filling and canal overfilling are significant risk factors for apical periodontitis [25,46], while the rate of apical periodontitis increases significantly in teeth with short root canal fillings when compared to teeth with proper root canal fillings [25,47,48].

In univariate analysis and multivariate analysis, we found poor coronal marginal sealing to be a risk factor for apical periodontitis both for the vital pulp group and teeth with necrotic pulp before the endodontic treatment. Multivariable multilevel logistic regression performed by Keratiotis et al. (2024) revealed that adequate density of the root canal fillings and coronal restorations with adequate quality reduced the likelihood of apical periodontitis [49]. Other previous studies found the coexistence of poor coronal restorations with poor root canal fillings to be a predictor of apical periodontitis [24,48,49,50,51,52,53,54]. AP was significantly associated with inadequacy of root canal filling but not with inadequacy of coronal restoration [55].

These results show that failure to achieve elimination of bacterial infection from the endodontic space, tooth cavities, failure to achieve asepsis during dental procedures, unacceptable root canal obturation, and re-infection because of unsound coronal restoration during or after root canal treatment are the main reasons for formation of periapical pathology.

The presence of caries adjacent to coronal restorations was found to be a risk factor for apical periodontitis in the root-filled teeth with vital pulp before endodontic therapy. This pathology is related both to patient cariogenic risk and poor coronal marginal sealing [56]. Various research groups have reported controversial data about the role of caries adjacent to coronal restorations in teeth with adequate root canal fillings. The presence of recurrent caries was not associated with apical periodontitis in one study [34], while another research group found recurrent caries to be a risk factor for endodontic treatment failure [48].

The presence of the periodontal pathology (BOP and PPD ≥ 3 mm) was a significant risk factor for endodontic failure in our study (OR 3.403 in univariate analysis, OR 3.334 in multivariate analysis). Periodontal pathology can pose significant additional risks for the longevity of affected teeth and can worsen a tooth’s overall long-term prognosis [57,58]. This can be remedied through thorough root debridement and non-surgical therapy and surgical complex therapy [59,60]. Adjunctive periodontal therapy, such as photo-disinfection, laser, application of local anti-inflammatory and antibiotic substances, etc., can also bring great benefits to patients with impaired wound healing such as smokers or patients with systemic conditions [61,62,63,64,65,66,67,68]. The periodontal–endodontic pathway can spread infection from periodontal pockets in apical areas in teeth with poor root canal fillings. Marginal bone loss was associated with apical periodontitis in root-filled teeth, while the rate of apical periodontitis was significantly lower in patients that had received periodontal treatment when compared with untreated periodontal patients [69,70,71]. Ruiz et al. observed that the risk of developing AP in endodontically treated teeth is 5.19 times higher for patients with periodontal disease compared with patients without periodontal disease. Severe periodontal disease was also a significant risk factor in a study that investigated the relation between sociodemographic/clinical factors and apical periodontitis [70]. Kato et al. (2020) found a significant relation between poor-quality root canal fillings, BOP, and periodontal pocket depth [72].

The presence of endo-periodontal lesions can worsen the prognosis of a previously correctly treated tooth, and the complex retreatment does not guarantee success [7]. The local and systemic inflammatory load is elevated in patients with endo-periodontal syndrome, especially in those with severe periodontitis. The authors state that these individuals have a much higher risk of developing and worsening of systemic maladies, such as diabetes mellitus, since local inflammation can affect the general inflammatory status [73].

If endodontic pathology reoccurs, loco-regional complications can appear, such as odontogenic sinusitis, thus leading to a more radical treatment plan and the extraction of the causing tooth [74].

Radiographic diagnostics had lower sensitivity in the diagnosis of early periapical lesions when compared to cone-beam CT in evaluation of periapical status [74,75,76,77,78,79]. The AI demonstrated high sensitivity and high specificity in detecting apical periodontitis in CBCT images but lower sensitivity in OPG images. [80]. Thus, root-filled teeth with incipient periapical lesions undetected in digital radiographic images were excluded from the study group.

While prospective studies can assess the effectiveness of irrigating solutions in biochemical and mechanical disinfection of the endodontic space, smear-layer removal, and the proper use of intracanal dressing, these are risk factors that cannot be assessed in longitudinal studies. Failure to achieve elimination of bacterial infection from the endodontic space during chemo-mechanical preparation as well as failure to achieve asepsis during dental procedures are correlated to the presence of apical periodontitis [45].

The use of a dam to limit microbial contamination is essential for successful endodontic treatment [81]. Also, the use of additional methods for disinfection of the endodontic canals, such as photoactivation or laser, has been proven useful and could be considered as a viable adjuvant therapy [82].

Our paper highlights some differences in AP prevalence and risk factors between root-filled teeth with vital pulp and necrotic pulp preoperatively. Thus, the higher prevalence of endodontic treatment failure in root-filled teeth treated for necrotic pulp proves that treatment should not be delayed in cases of irreversible pulpal disease or necrotic pulp, as timely intervention is more effective in preventing rather than resolving apical periodontitis. Also, the clinical approach for these two conditions must adhere to standard guidelines to prevent apical periodontitis by maintaining an aseptic technique and avoiding infection through coronal leakage [2].

Some limitations of the study are the more ample division of possible therapeutic situations, the use of CBCT at baseline and retreatment, the use of AI in the interpretation of radiographic images, and the more equal distribution of vital pulp teeth and necrotic pulp teeth (at initial diagnosis).

Further studies must also investigate other factors such as the enlargement of the apical foramen, zips, ledges, perforations, and transport of the root canals.

## 5. Conclusions

Most cases of apical periodontitis were associated with inadequate root canal filling and periodontitis in root-filled teeth with vital and necrotic pulp. Other significant risk factors include an age > 60 yrs., poor coronal restoration quality, and the presence of intracanal posts in root-filled teeth with necrotic pulp. The analysis of patient risk factors in the planning of the endodontic treatment can decrease the burden of apical periodontitis. Care should be taken concerning the quality of root canal fillings, the seal of the coronal cavity, and periodontal infection control.

## Figures and Tables

**Table 1:** Description of the studied population.

Parameter	Vital Pulp		Necrotic Pulp		Total		*p*-Value
	*n*	%	*n*	%	*n*	%	
*Global*	52	100.0	99	100.0	151	100.0	
*Gender*							0.295
M	15	28.8	37	37.4	52	34.4	
F	37	71.2	62	62.6	99	65.6	
*Age (yrs.)* (mean, SD)	Globally: 52.81 ± 18.647 M: 48.67 ± 24.839/F: 54.49 ± 15.577 *p* = 0.316		Globally: 46.02 ± 12.326 M: 42.27 ± 12.055/F: 48.26 ± 12.030 *p* = 0.116		Globally: 48.36 ± 15.098 M: 44.12 ± 16.748/F: 50.59 ± 13.725 *p* = 0.012 *		0.042 *
*Age groups (yrs.)*							<0.001 **
20–39	13	25.0	28	28.3	41	27.2	
40–59	21	40.4	63	63.6	84	55.6	
≥60	18	34.6	8	8.1	26	17.2	

* Statistically significant. ** Highly statistically significant.

**Table 2:** Description of the study group.

Parameter	Vital Pulp		Necrotic Pulp		Total		*p*-Value
	*n*	%	*n*	%	*n*	%	
*Global*	127	100.0	264	100.0	391	100.0	
*Gender*							0.181
M	43	33.9	72	27.3	115	29.4	
F	84	66.1	192	72.7	276	70.6	
*Age*							<0.001 **
20–39 yrs.	32	25.2	50	18.9	82	21.0	
40–59 yrs.	43	33.9	192	72.7	235	60.1	
≥60 yrs.	52	40.9	22	8.3	74	18.9	
*Follow-up (yrs.)* *(mean ± SD)*	5.16 ± 1.716		5.29 ± 1.781		5.25 ± 1.759		0.863
*Tooth type*							0.905
Anterior	47	37.0	103	39.0	150	38.4	
Premolar	38	29.9	79	29.9	117	29.9	
Molar	42	33.1	82	31.1	124	31.7	
*Location* (*MX*/*MD*)							0.955
MX	79	62.2	165	62.5	244	62.4	
MD	48	37.8	99	37.5	147	37.6	
*Dental crown restoration*							
Composite direct restoration	20	15.7	72	27.3	92	23.5	0.012 *
Class Black I	10	50.0	20	27.8	30	7.7	0.158
Class Black II	8	40.0	44	61.1	52	13.3	
Class Black III	2	10.0	4	5.6	6	1.5	
Class Black IV	-	-	4	5.6	4	1.0	
Complete coverage crown	107	84.3	192	72.7	299	76.5	
Intracanal posts	14	11.0	95	36.0	109	27.9	<0.001 **
*Coronal restoration quality*							0.056
Adequate	57	44.9	92	34.8	149	38.1	
Poor	70	55.1	172	65.2	242	61.9	
*Root canal filling status*							<0.001 **
Proper	42	33.1	42	15.9	84	21.5	
Poor	85	66.9	222	84.1	307	78.5	
Short filling	65	51.2	159	60.2	224	57.3	0.090
Canal overfilling	14	11.0	19	7.2	33	8.4	0.202
Poor homogeneity	65	51.2	200	75.8	265	67.8	<0.001 **
Inadequate taper (conicity)	67	52.8	192	72.7	259	66.2	<0.001 **
*Antagonist tooth*							0.158
YES	99	78.0	188	71.2	287	73.4	
NO	28	22.0	76	28.8	104	26.6	
*Caries adjacent to restoration*							0.028 *
YES	66	52.0	168	63.6	234	59.8	
NO	61	48.0	96	36.4	157	40.2	
*Periodontal pathology*							0.004 **
Absent	54	42.5	153	58.0	207	52.9	
Present (BOP, PPD ≥ 3 mm)	73	57.5	111	42.0	184	47.1	
*Endo-periodontal lesions*							0.510
YES	2	1.6	8	3.0	10	2.6	
NO	125	98.4	256	97.0	381	97.4	
Periapical status							<0.001 **
Healthy	92	72.4	118	44.7	210	53.7	
Apical periodontitis	35	27.6	146	55.3	181	46.3	

* Statistically significant; ** Highly statistically significant.

**Table 3:** Criteria for the assessment of root canal fillings and coronal restorations [25,31].

Parameters of the Root Canal Fillings	Criteria	Description
Density	Adequate	Gaps are absent in the canal root filling or at the interface between the root filling and root canal walls
	Poor	Gaps are present in the canal root filling or at the interface between the root filling and root canal walls
Length	Adequate	Root filling ends below 2 mm from the radiographic apex
	Poor	Root filling extends over the radiographic apex or ends > 2 mm from the radiographic apex
Taper (conicity)	Adequate	Root canal preparation is continuously tapered, being funneled from the canal entrance to the apex, while the cross-sectional diameter is narrower at every point apically
	Poor	Taper (conicity) is inconsistent from the canal entrance to the apical part, or root filling was deviated from the original canal
Parameters of the tooth crown restoration		
	Adequate	Permanent direct or indirect restoration with an intact radiographic aspect
	Poor	Permanent direct or indirect restoration with visible signs of caries adjacent to restoration, open margins, or the presence of temporary coronal restoration
	Missing	Absence of direct or indirect coronal restoration

**Table 4:** Clinical and radiographic success/failure (absence/presence of apical periodontitis) according to assessed variables.

Group A—VITAL PULP	AP				*p*-Value *
Parameter	Present		Absent		
	*N*	%	*N*	%	
*Gender*					0.232
M	9	20.9	34	79.1	
F	26	31.0	58	69.0	
*Age*					0.795
20–39 yrs.	8	25.0	24	75.0	
40–59 yrs.	11	25.6	32	74.4	
≥60 yrs.	16	30.8	36	69.2	
*Follow-up*					0.238
2–4 yrs.	12	35.3	22	64.7	
5–8 yrs	23	24.7	70	75.3	
*Tooth type*					0.111
Anterior	17	36.2	30	63.8	
Premolar	6	15.8	32	84.2	
Molar	12	28.6	30	71.4	
*Location* (*MX*/*MD*)					0.752
MX	21	26.6	58	73.4	
MD	14	29.2	34	70.8	
*Dental crown restoration*					0.014 *
Composite direct restoration	10	50.0	10	50.0	
Complete coverage crown	25	23.4	82	76.6	
*Composite direct restoration*					0.111
Class Black I	6	60.0	4	40.0	
Class Black II	2	25.0	6	75.0	
Class Black III	2	100.0	-	-	
Class Black IV	-	-	-	-	
Intracanal posts	2	14.3	12	85.7	0.347
*Coronal marginal sealing*					0.007 **
Adequate	9	15.8	48	84.2	
Poor	26	37.1	44	62.9	
*Root canal filling status*					<0.001 **
Proper	-	-	42	100.0	
Poor	35	41.2	50	58.8	
Short filling	23	35.4	42	64.6	0.043 *
Canal overfilling	10	71.4	4	28.6	<0.001 **
Poor homogeneity	33	50.8	32	49.2	<0.001 **
Inadequate taper (conicity)	29	43.3	38	56.7	<0.001 **
*Antagonist tooth*					0.040 *
YES	23	23.2	76	76.8	
NO	12	42.9	16	57.1	
*Caries adjacent to restoration*					<0.001 **
YES	28	42.4	38	57.6	
NO	7	11.5	54	88.5	
*Periodontal pathology*					<0.001 **
Absent	4	7.4	50	92.6	
Present (BOP, PPD ≥3 mm)	31	42.5	42	57.5	
*Endo-periodontal lesions*					0.074
YES	2	100.0	-	-	
NO	33	26.4	92	73.6	
**Group B—** **NECROTIC PULP**	**PAC**				*p*-value *
**Parameter**	**Present**		**Absent**		
	***N***	**%**	***N***	**%**	
*Gender*					0.613
M	38	52.8	34	47.2	
F	108	56.3	84	43.8	
*Age*					0.024 *
20–39 yrs.	24	48.0	26	52.0	
40–59 yrs.	104	54.2	88	45.8	
≥60 yrs.	18	81.8	4	18.2	
*Follow-up*					0.072
2–4 yrs	68	61.8	42	38.2	
5–8 yrs	78	50.6	76	49.4	
*Tooth type*					0.046 *
Anterior	59	57.3	44	42.7	
Premolar	35	44.3	44	55.7	
Molar	52	63.4	30	36.6	
*Location (MX/MD)*					0.110
MX	85	51.5	80	48.5	
MD	61	61.6	38	38.4	
*Coronal restoration type*					0.289
Composite direct restoration	36	50.0	36	50.0	
Complete coverage crown	110	57.3	82	42.7	
*Direct composite restoration*					0.225
Class Black I	10	50.0	10	50.0	
Class Black II	24	54.5	20	45.5	
Class Black III	2	50.0	2	50.0	
Class Black IV	-	-	4	100.0	
Intracanal posts	63	66.3	32	33.7	0.007 **
*Coronal marginal sealing*					<0.001**
Adequate	36	39.1	56	60.9	
Poor	110	64.0	62	36.0	
*Root canal filling status*					<0.001 **
Proper	12	28.6	30	71.4	
Poor	134	60.4	88	39.6	
Short filling	101	63.5	58	36.5	<0.001 **
Canal overfilling	19	100.0	-	-	<0.001 **
Poor homogeneity	126	63.0	74	37.0	<0.001 **
Inadequate taper (conicity)	122	63.5	70	36.5	<0.001 **
*Antagonist tooth*					0.579
YES	106	56.4	82	43.6	
NO	40	52.6	36	47.4	
*Caries adjacent to restoration*					0.019 *
YES	102	60.7	66	39.3	
NO	44	45.8	52	54.2	
*Periodontal pathology*					<0.001 **
Absent	63	41.2	90	58.8	
Present (BOP, PPD ≥ 3 mm)	83	74.8	28	25.2	
*Endo-periodontal lesions*					0.009 **
YES	8	100.0	-	-	
NO	138	53.9	118	46.1	

* Pearson Chi-squared test/Fisher’s exact test.; ** Highly statistically significant.

**Table 5:** Univariate and multivariate logistic regression for the association of study variables with periapical status. (prevalence of AP and OR in relation to the assessed parameters).

Group A—VITAL PULP Parameter	Univariate Analysis			Multivariate Analysis		
	OR	95% CI	*p*-Value	OR	95% CI	*p*-Value
*Dental crown restoration*						
Composite direct restoration	1					
Complete coverage crown	3.280	1.226 ÷ 8.777	0.014 *	0.000	-	0.990
*Coronal marginal sealing*						
Adequate	1					
Poor	3.152	1.332 ÷ 7.458	0.007 **	0.000	-	0.989
*Root canal filling status*	-					
Proper						
Poor		-	<0.001 **	0.147	-	1.000
Short filling	2.282	1.015 ÷ 5.127	0.043 *	3.185 × 10^14^	-	0.999
Canal overfilling	8.800	2.542 ÷ 30.461	<0.001 **	1.800 × 10^31^	-	0.999
Poor homogeneity	30.938	6.970 ÷ 137.324	<0.001 **	5.082 × 10^50^	-	0.989
Inadequate taper (conicity)	6.868	2.598 ÷ 18.156	<0.001 **	0.000	-	0.999
*Antagonist tooth*						
NO	1					
YES	2.478	1.026 ÷ 5.986	0.040 *	3.554 × 10^7^	-	0.995
*Caries adjacent to restoration*						
Absent	1					
Present	5.684	2.251 ÷ 14.355	<0.001 **	0.000	-	0.996
*Periodontal pathology*						
Absent	1					
Present (BOP, PPD ≥ 3 mm)	9.226	3.013 ÷ 28.254	<0.001 **	3.873 × 10^43^	-	0.989
**Group B—NECROTIC PULP** **Parameter**	**Univariate analysis**			**Multivariate analysis**		
	**OR**	**95% CI**	***p*-value**	**OR**	**95% CI**	***p*-value**
*Age*						
20–39 yrs.	1			1		0.047 *
40–59 yrs.	1.280	0.687 ÷ 2.388	0.437	2.159	0.856 ÷ 5.442	0.103
≥60 yrs.	4.875	1.443 ÷ 16.466	0.007 **	6.464	1.423 ÷ 29.365	0.016 *
*Tooth type*						
Premolar	1			1		0.014 *
Anterior	1.686	0.933 ÷ 3.045	0.082	1.834	0.874 ÷ 3.847	0.109
Molar	2.179	1.159 ÷ 4.098	0.015 *	3.583	1.514 ÷ 8.476	0.004 **
Intracanal posts	2.040	1.211 ÷ 3.436	0.007 **	2.023	1.010 ÷ 4.054	0.047 *
*Coronal marginal sealing*						
Adequate	1			1		
Poor	2.760	1.638 ÷ 4.650	<0.001 **	2.684	1.096 ÷ 6.572	0.031 *
*Root canal filling status*						
Proper	1			1		
Poor	3.807	1.850 ÷ 7.832	<0.001 **	1.583	0.407 ÷ 6.161	0.508
Short filling	2.322	1.403 ÷ 3.842	<0.001 **	3.578	1.406 ÷ 9.107	0.007 **
Canal overfilling	-	-	<0.001 **	4.313 × 10^9^	-	0.998
Poor homogeneity	3.746	2.053 ÷ 6.836	<0.001 **	1.800	0.515 ÷ 6.294	0.358
Inadequate taper (conicity)	3.486	1.969 ÷ 6.172	<0.001 **	0.358	0.095 ÷ 1.356	0.131
*Caries adjacent to restoration*						
Absent	1					
Present	1.826	1.100 ÷ 3.032	0.019 *	0.440	0.168 ÷ 1.149	0.094
*Periodontal pathology*						
Absent	1					
Present (BOP, PPD ≥ 3 mm)	4.235	2.478 ÷ 7.237	<0.001 **	4.372	1.942 ÷ 9.843	<0.001 **
*Endo-periodontal lesions*						
NO						
YES	-	-	0.009 **	1.696 × 10^8^	0.000	0.999

* Statistically significant; ** Highly statistically significant.

## Data Availability

Data supporting reported results can be provided by the corresponding authors upon reasonable request.
